# Redefining Hormone Sensitive Disease in Advanced Prostate Cancer

**DOI:** 10.1155/2012/978531

**Published:** 2012-02-25

**Authors:** Xiaoyu Hou, Thomas W. Flaig

**Affiliations:** Division of Medical Oncology, University of Colorado Denver School of Medicine, 12801 E. 17th Avenue, Room L18-8117, Aurora, CO 80045, USA

## Abstract

Prostate cancer is the most common cancer among men in the United States. For decades, the cornerstone of medical treatment for advanced prostate cancer has been hormonal therapy, intended to lower testosterone levels, known as Androgen Deprivation Therapy (ADT). The development of hormone-resistant prostate cancer (now termed castration-resistant prostate cancer:CRPC) remains the key roadblock in successful long-term management of prostate cancer. New advancements in medical therapy for prostate cancer have added to the hormonal therapy armamentarium. These new therapeutic agents not only provide a survival benefit but also show potential for reversing hormonal resistance in metastatic CRPC, and thus redefining hormonally sensitive disease.

## 1. Background

Prostate cancer is the 2nd most frequently diagnosed cancer and the 6th leading cause of death among males worldwide [1]. In 2011, it is estimated that there will be 240,890 new cases of prostate cancer and an estimated 33,720 deaths due to prostate cancer in the USA [2]. A variety of primary treatment modalities exist to treat localized cancer of the prostate including surgery, external beam radiation therapy, brachytherapy, cryosurgery, and High Intensity Focused Ultrasound (HIFU). However, for metastatic prostate cancer, the initial treatment has traditionally been hormonal therapy. ADT is effective for 2-3 years on average, and when hormonal therapy fails, chemotherapy provides a survival and palliative benefit, at the cost of considerable side effects. ADT has been given standalone and as an adjunct with other treatment modalities such as chemotherapy, radiation, and surgery [3]. 

## 2. History of Androgen Deprivation Therapy in Prostate Cancer

Hormonal therapy has long been an integral part of prostate cancer treatment. In 1811, Scottish surgeon John Hunter (1728–1793) observed the relationship between prostate growth and testicular function for the first time in his textbook *Practical Observations on the Treatment of Diseases of the Prostate Gland *[4]. Louis Auguste Mercier (1811–1882) of France first performed orchiectomy for the treatment of an enlarged prostate in 1857 [4]. In 1941, Huggins first used estrogen to treat metastatic prostate cancer, which led to a Nobel prize in 1966, representing one of the first successful systemic therapies for cancer [5].

Similar to breast cancer, prostate cancer is a hormonally driven solid malignancy. Androgens are the key driver of growth for both the normal prostate and prostate cancer cells. Vital in the definition of castrate-resistant disease is the recognition of the potential sources of androgen and approaches aimed at the reduction of their levels. While we have historically focused on the testicular production of androgens, other sites of production include the adrenal glands and the more recently appreciated source of intratumoral androgen production. Normally 90–95% of circulating testosterone is produced by the Leydig cells of the testes. Another 5–10% of systemic testosterone is synthesized by the adrenal glands [6]. While production by the testis is the main source of androgen in prostate cancer prior to castration, it has now been appreciated that traditionally defined CRPC is frequently driven by intratumoral androgen production and synthesis of testosterone from weak adrenal androgen precursors [7, 8].

Given the multiple sources and associated biosynthesis pathways of androgen productions, hormonal therapy in prostate cancer is achieved through multiple mechanisms with several different classes of agents (Table 1).

**Figure 1:**
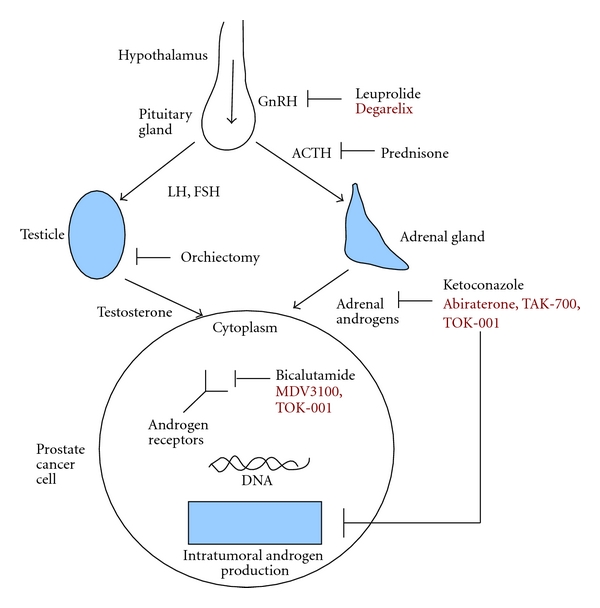
Hormonal therapy in prostate cancer. Physiologically relevant androgens for prostate cancer originate from three sources: the testicle, adrenal glands, and via intratumoral production. The sites of activity of clinically relevant hormonal therapies are illustrated here, with new and investigational treatments indicated in red.

Though in limited use today, estrogen was initially used to systematically treat prostate cancer decades ago. Beyond an agent simply to induce a reduction in testosterone levels, Serrate et al. reported in 2009 that diethylstilbestrol (DES) is a reasonable option for salvage therapy for CRPC in patients previously treated with docetaxel chemotherapy, suggesting a direct anticancer effect and larger “hormone sensitive” treatment window [9]. Surgical orchiectomy remains a viable option to achieve androgen deprivation to this day [10]. Medical castration can be achieved by using gonadotropin-releasing hormone (GnRH) agonists such as goserelin, leuprolide, and histrelin acetate, which induce androgen deprivation through persistent overstimulation and subsequent downregulation at the level of the GnRH receptor [11, 12]. GnRH antagonists such as degarelix can also disrupt androgen production and are a more recent addition to our medical treatment options [13]. Antiandrogens such as bicalutamide, nilutamide, and flutamide block the effect of androgen directly at the androgen receptor, although the blockade of the androgen receptor is incomplete and partial agonist properties are observed with these agents [14]. In contrast, MDV3100 is a new generation antiandrogen with great affinity for the androgen receptor and no known agonist effect [15]. Ketoconazole is a nonspecific inhibitor of cytochrome P450 enzyme-mediated androgen biosynthesis [16]. Glucocorticoids such as prednisone have been used as a palliative agent and likely act by suppressing ACTH secretion and thus reducing adrenal androgen production [17] (Figure 1).

Common side effects of hormonal therapy in prostate cancer treatment include hot flashes, weight gain, gynecomastia, and osteoporosis [18]. Estrogen additionally has cardiovascular side effects including blood clots [19] and there is also evidence of increased cardiovascular risk with GnRH agonist in this setting [20]. Side effects of ketoconazole include elevated liver transaminases and gastrointestinal complaints [21].

Hormonal therapy can produce dramatic clinical responses when initially used to treat advanced prostate cancer. Unfortunately, prostate cancer patients become resistant to androgen deprivation after about 2-3 years on average with progressive disease, despite castrate levels of testosterone [22, 23]. It is hypothesized that despite reduced androgen levels, one mechanism of resistance is the production of androgen for growth via intratumoral and extragonadal pathways. Other mechanisms of hormonal resistance include upregulation of androgen receptors with increased sensitivity to androgen at the androgen receptors, and mutations of androgen receptors itself [24, 25].

## 3. Current Advancement in Hormonal Therapy

Historically, a serum testosterone level of <50 ng/dL (<1.74 nmol/L) has been used as the benchmark to assess the efficiency of hormonal therapy, as comparable to the level of suppression achieved with surgical castration [26]. Subsequently, disease progression after achieving castration levels of serum testosterone has been used as the definition of CRPC. For many years, clinicians lacked rigorously proven therapeutic hormonal options for the treatment of prostate cancer after the development of castration-resistant disease, with only docetaxel chemotherapy providing a clear survival benefit in this setting [27, 28]. However, the introduction of several new hormonal agents has challenged the traditional definition of CRPC.

The CYP17 enzyme is a member of the cytochrome P450 family of enzymes that mediates the biosynthesis of dehydroepiandrosterone and androstenedione, both precursors of testosterone. Previously, nonselective inhibitors of CYP17 such as ketoconazole have been used in prostate cancer treatment [29]. In contrast, abiraterone acetate has been developed to selectively and irreversibly inhibit the CYP17 enzyme (17*α*-hydroxylase and C17,20-lyase) [30] and demonstrated significant antitumor activities against prostate cancer in phase I/II clinical trials [31, 32]. Similar to abiraterone, TAK-700 (Orteronel) is an investigational CYP17 inhibitor, that may offer a more precise targeting of the CYP17 biosynthesis pathway by inhibiting only C17,20-lyase [33]. Clinical trials of TAK-700 are currently ongoing [34]. TOK-001 (Galeterone) is another promising investigational CYP17 inhibitor that is currently being evaluated a phase I/II clinical trial (ARMOR1: Androgen Receptor Modulation Optimized for Response 1) [35]. Uniquely, TOK-001 is not only a selective CYP17 (17*α*-hydroxylase and C17,20-lyase) inhibitor analogous to abiraterone acetate but also an androgen receptor modulator [36]. These new selective CYP17 enzyme inhibitors represent an important bench-to-bedside advancement, responding to the need for more potent and specific inhibitors of extra-gonadal androgen production.

In a landmark phase III clinical trial from 2011, de Bono et al. reported that abiraterone acetate plus prednisone compared to prednisone alone yielded an improved median overall survival from 10.4 to 14.8 months in patients with metastatic hormonal-resistant prostate cancer after docetaxel chemotherapy (*P* < 0.0001). Circulating serum testosterone levels are reduced to levels of 1-2 ng/dL with abiraterone acetate—much lower than the previous standard of 50 ng/dL used to define CRPC. In this trial, abiraterone plus prednisone also showed significant improvement in time to PSA progression (10.2 months versus 6.6 months; *P* < 0.0001), radiographic progression-free survival (PFS) (5.6 months versus 3.6 months; *P* < 0.0001), and PSA response rate (29.1% versus 5.5%; *P* < 0.0001) when compared to prednisone alone. The immediate side effects of abiraterone were manageable and primarily related to elevated mineralocorticoid levels, including hypertension, fluid retention, and hypokalemia [37].

While androgen receptor signaling remains an important pathway of growth in CRPC, the currently available antiandrogens have not been able to completely block androgen receptor signaling [38]. In another area of bench-to-bedside advancement, a new generation antiandrogen (MDV3100) is now in clinical development with encouraging results. Unlike currently available antiandrogens, MDV3100 is a pure androgen receptor antagonist without agonist activity. It is differentiated from the current antiandrogens by its more effective blocking of androgen receptor nuclear translocation and coactivator recruitment of the ligand-receptor complex [39]. In a multicenter, phase I/II study involving 140 patients from 2010, Scher et al. reported that MDV3100 showed antitumor activities in patients with metastatic CRPC, including decreases in serum prostate-specific antigen of 50% or more in 78 of 140 (56%) patients, responses in soft tissue in 13 of 59 (22%) patients, stabilized bone disease in 61 of 109 (56%) patients, and conversion from unfavorable to favorable circulating tumor cell counts in 25 of 51 (49%) patients [40]. Side effects include fatigue, which generally resolved with dose reduction [40] and seizure at higher doses [41]. Phase III studies examining the efficacy of MDV3100 both before and after docetaxel chemotherapy in men with CRPC are underway [34, 42].

GnRH plays a key role in the androgen axis. GnRH agonists have been used to achieve medical castration in prostate cancer for decades. Unlike the original GnRH receptor agonists, the recently approved degarelix acts as a direct GnRH antagonist, suppressing LH release without the iniital androgen flare and potential exacerbation of symptoms noted with GnRH receptor agonists [43]. GnRH antagonists such as degarelix also have a faster onset of action with less delay in the suppression of testosterone to castrate levels than GnRH agonists [44]. In a phase III clinical trial from 2008, Klotz et al. reported that when compared to leuprolide after a 1-year treatment period, degarelix was not inferior to leuprolide at maintaining low testosterone levels. In addition, degarelix achieved testosterone and PSA suppression more rapidly than leuprolide with no need for antiandrogen supplements to prevent the initial clinical flare. Manageable side effects of degarelix include flushing, injection-site pain, weight gain, and increased serum transaminase levels [45]. It is important to highlight that when compared to leuprolide, degarelix might offer better suppression of serum alkaline phosphatase (S-ALP) level and more prolonged control of skeletal metastases [46]. Furthermore, though degarelix and leuprolide both act on the GnRH receptor, reports of response to degarelix after previous resistance to GnRH agonist therapy with leuprolide have appeared [47], also potentially complicating our traditional definition of CRPC.

## 4. Discussion

The phase III, landmark findings of the abiraterone acetate study by de Bono et al. have drawn into question our traditional definition of CRPC. Previously, phase I/II clinical trials of the CYP17 inhibitor abiraterone acetate demonstrated clinical activity in CRPC [31, 32]. This phase III study demonstrates a clear survival benefit from additional hormonal manipulation in a prostate cancer population previously described as hormone refractory. Clearly, simply defining CRPC as progressive disease with a serum testosterone level of less than 50 ng/dL is no longer adequate. The findings of de Bono et al. are consistent with other investigations showing ongoing androgen-related activity in the post-chemotherapy setting. Going forward, the definition of hormone-sensitive prostate cancer will need to incorporate the use of a potent CYP17 enzyme inhibitor such as abiraterone acetate. Accordingly, a much lower level of systemic testosterone, beyond the traditional 50 ng/dL benchmark and closer to the 1-2 ng/dL level, must be targeted to achieve “complete” androgen deprivation. New investigational agents such as TAK-700 and TOK-001 represent addition agents in this class of CYP17 inhibitors, currently undergoing clinical testing in CRPC [33, 35]. Additionally, the use of new generation antiandrogens, such as MDV3100, must also be integrated into our definition of hormone-sensitive prostate cancer. The available clinical trial data with this drug, although early and limited, suggests substantial activity in a traditionally defined CRPC population [48].

It is exciting to envision a future with additional clinical gains from the earlier use of potent CYP17 enzyme inhibitors in the course of disease treatment or with combination therapy of abiraterone acetate and other newer agents such as MDV3100. As a general principle, selective pressures from cancer therapy typically yield resistant strains of disease with progressively more aggressive features. Currently, we do not have a good understanding of the phenotypes of advanced prostate cancer that may emerge after early treatment with much more potent hormonal therapies such as MDV3100 and abiraterone acetate. Many unanswered questions remain about the prostate cancer phenotype that will emerge after early use of CYP17 inhibition in terms of robustness, virulence, speed of disease progression, and the responsiveness to cytotoxic chemotherapy.

Since John Hunter first described the link between the testis and the prostate over 200 years ago, hormonal therapy remains one of the mainstays of advanced prostate cancer treatment. After initial response, metastatic prostate cancer typically becomes resistant to standard androgen deprivation therapy. Improved survival in this setting has been demonstrated in recent clinical trials involving new agents such as the CYP17 inhibitor abiraterone acetate and the antiandrogen MDV3100, showing that CRPC remains hormonally driven even after treatment with docetaxel chemotherapy, challenging our traditional definition of hormone-sensitive disease. Even though the survival benefit is incremental in the phase III study of very advanced CRPC patients, a threshold has clearly been crossed in defining and treating advanced prostate cancer.

With so many new agents for advanced prostate cancer, individual patient characteristics may become very important in the selection and monitoring of therapy. The advantages of Degarelix over previous GnRH agonists may be incremental for most CRPC patients, but the quicker fall in testosterone may be very important to those with critical metastatic or painful lesions such as spinal metastases or near-total urinary obstruction. Likewise, the combination of “complete” androgen deprivation with abiraterone along with the required safety medication of low-dose glucocorticoid may induce more bone disease via worsening osteoporosis. Men with preexisting bone mineral density loss in this setting may require careful monitoring of the bone mineral density and the use of early bisphosphonates. In this same light, the side effect profile of a potent antiandrogen, which will not lower circulating androgen levels by itself, may become an important consideration if monotherapy with such agents becomes accepted. Furthermore, much like breast cancer is classified and treated based on hormonal receptor characterization today, we believe that advanced prostate cancer may one day also be treated based on molecular assessment. As more is known about the molecular characterization of advanced prostate cancer, it will be imperative to develop more personalized hormonal therapy for individual patients. Taken together, one of the major challenges moving forward will be the personalized application of these new agents in those with advanced CRPC, now that we have many more hormonal agents at our disposal with different side effects and mechanisms of action.

## Figures and Tables

**Table 1:** Hormonal therapy in prostate cancer.

Class	Existing drugs	Investigational drugs
Estrogen	Diethylstilbestrol (DES)	
GnRH agonists	Goserelin, leuprolide, triptorelin, histrelin acetate	
GnRH antagonists	Degarelix, *abarelix	
Antiandrogens	Bicalutamide, nilutamide, flutamide, **cyproterone acetate	MDV3100, TOK-001
Non-specific,cytochrome P450 enzyme inhibitors	Ketoconazole	
Specific CYP17 inhibitors	Abiraterone acetate	TAK-700, TOK-001
Glucocorticoids	Prednisone, Dexamethasone, others

*Abarelix use in the United States was previously limited to a registry program and it is not actively marketed in the United States currently.

**Cyproterone acetate is not currently FDA approved for the treatment of prostate cancer in the United States.
